# Potential clinical utility of plasma D-dimer levels among women with cervical cancer in Lagos, Nigeria

**DOI:** 10.3332/ecancer.2023.1501

**Published:** 2023-01-30

**Authors:** Lucky E Tietie, Kehinde S Okunade, Adaiah P SoibI-Harry, Sarah O John-Olabode, Rose I Anorlu

**Affiliations:** 1 Oncology and Pathological Studies Unit, Lagos University Teaching Hospital, Lagos 102215, Nigeria; 2 Department of Obstetrics & Gynaecology, College of Medicine, University of Lagos, PMB 12003, Lagos, Nigeria; 3 Department of Haematology and Blood Transfusion, College of Medicine, University of Lagos, PMB 12003, Lagos, Nigeria

**Keywords:** FIGO stage, cervical malignancy, marker, ROC, Lagos

## Abstract

The link between plasma D-dimer levels and underlying malignancy has been established. How this translates in clinical practice as a marker of detection and prognosis of cervical cancer (CC) is still unknown. This study compared the plasma D-dimer levels in women with and without CC and assessed the associations between plasma D-dimer levels and the stage and grade of CC. It was a comparative cross-sectional study of 65 women with histological diagnosis of CC and an equal number of age-matched cancer-free women enrolled at the University Teaching Hospital in Lagos, Nigeria. Participants’ sociodemographic and clinical data as well as venous blood samples for estimation of plasma D-dimer were collected for statistical analyses. A receiver operating characteristic (ROC) analysis is performed to select the cut-off value of plasma D-dimer for differentiating CC from non-cancer. There was a statistically significant difference in the median levels of plasma D-dimer of women with CC and their cancer-free comparison groups (3,120 (1,189–4,515) versus 210 (125–350) ng/mL; *p* = 0.001). A plasma D-dimer value of 543 ng/mL was chosen in a ROC analysis as the discriminatory cut-off to differentiate CC from non-cancer. There were significant associations between plasma D-dimer levels and the International Federation of Gynaecology and Obstetrics stage (*p* = 0.001) or grade (*p* = 0.001) of CC. The study, therefore, demonstrated the potential clinical usefulness of plasma D-dimer as a diagnostic and prognostic marker of CC.

## Introduction

Cervical cancer (CC) constitutes a significant public health burden in many resource-limited countries [1]. In 2020, it accounted globally for an estimated 604,000 new cases and 342,000 deaths with Africa bearing the largest burden of the disease [2]. Nigeria accounts for 14,943 new cases and 10,403 cancer-related deaths annually [3]. Many biomarkers have been previously used to screen for CC and assess the risk of recurrence after treatment. However, these biomarkers are not sufficient to predict prognosis accurately and their clinical usefulness is still being debated [4]. Therefore, the search for a more reliable marker of early detection and prognostic monitoring of CC continues. Serum tumour biomarkers have been shown to play several roles in cancer management including early detection through screening, diagnostic confirmation, prognostication, monitoring and response to specific treatments [5].

Activation of clotting is common in cancers; hence, there is a high incidence of thrombosis in metastatic, fast-growing, biologically aggressive cancer [6]. This activation also leads to the generation of D-dimer through the degradation of cross-linked fibrin resulting from the proteolytic actions of plasmin [6]. D-dimer is an exceptional marker of fibrin degradation that is formed because of the sequential activation of thrombin, factor XIIIa and plasmin in the coagulation cascade [7]. Elevated levels signal the occurrence of hyperfibrinolysis, and its clinical use is well established in excluding the diagnosis of venous thromboembolism (VTE), for making a diagnosis of acute aortic dissection and for risk stratification of patients for VTE recurrence [7]. Coagulation indexes including plasma D-dimer may be useful as survival biomarkers for several solid malignancies including CC [8]. High pretreatment plasma levels of D-dimer are frequently detected in patients with CC [9, 10], however, the prognostic significance of this finding is still largely unknown. Furthermore, there are still limited data available to show the association between plasma D-dimer levels and invasive CC among Nigerian women. This current study, therefore, evaluated the clinical significance of D-dimer levels in women with CC by comparing the plasma D-dimer levels of women with CC with that of a comparative cancer-free group of women and, in addition, determined the associations between plasma D-dimer levels and the clinical markers of prognosis such as the stage and grade of CC.

## Materials and methods

### Study design and setting

The study was a comparative cross-sectional study conducted among women diagnosed with CC and their healthy cancer-free counterparts enrolled at the gynaecological outpatient and radiation oncology clinics of the University Teaching Hospital in Lagos, Nigeria. The hospital is the teaching hospital of a College of Medicine that serves mainly as a referral centre for other government-owned and private hospitals in the state, and its environs.

### Study population and sample size determination

The study population included treatment-naïve (yet to commence any form of treatment) women with histologically diagnosed CC enrolled at the gynaecological outpatient and radiation oncology clinics and their age-matched comparison group comprising cancer-free women attending the gynaecological outpatient clinics for infertility treatment. All women with obesity (body mass index up to 30.0 kg/m^2^), those with major medical conditions such as hypertension, diabetes, liver or renal diseases, previous or concomitant history of cancer, haematological diseases, coagulation disorders or women on anticoagulants were excluded from this study. The sample size for each study group (*n* = 65) was estimated using the formula for comparison of two independent groups [11] to achieve power (1–Zβ) of 80% (0.842) at a type 1 error (Zα) rate of 5% (1.96) with 95% confidence level and effect size of 0.50 while adjusting for a non-response rate of 20%.

### Participant enrolment and data collection

Eligible participants for the study were enrolled by consecutive sampling until the required sample size was achieved. The purpose and procedures of the study were explained to all participants and their informed consent was obtained before enrolment in the study. A structured interviewer-administered questionnaire and patients’ medical records were used to obtain information such as socio-demographic characteristics, medical and reproductive history, details of cancer diagnosis, as well as the revised 2019 International Federation of Gynaecology and Obstetrics (FIGO) clinicopathologic stage (stage I to IV) [12] and the traditional Broders’ grading system of squamous cell carcinoma (SCC) [13] characterised by squamous differentiation – well-differentiated (low grade, G1), moderately differentiated (intermediate grade, G2), poorly differentiated (high grade, G3) and undifferentiated (high grade, G4) tumour [13]. Following this, about three millilitres (3 mL) of whole blood were collected from each participant’s antecubital vein, dispensed into a trisodium citrate anticoagulated vacutainer bottle labelled with the participant’s identification (ID) code, and then transported within 30 minutes to the departmental research laboratory where the specimen was centrifuged (at 1500 × g for 15 minutes) within 1 hour to avoid degradation. Following this, about 1 mL of platelet-poor plasma was extracted and stored at minus 20°C in cryogenic vials until laboratory analysis.

### Laboratory analysis

Plasma D-dimer concentration was determined using the fluorescence immunoassay rapid quantitative test that uses a sandwich immuno-detection method with an assay working range of 50~10,000 ng/mL and a detection limit of 50 ng/mL. About 10 µL of the plasma was drawn using a plastic micropipette for transfer and mixing in a detection buffer tube labelled with the participant’s ID code. Approximately 75 µL of the buffered mixture is then transferred in a micropipette into the sample well of the test cartridge. The fluorescence-labelled detector D-dimer antibodies on the sample pad bind to D-dimer antigens in the buffered blood specimen in the test cartridge to form immune complexes in a quick test mode. The complexes migrate by capillary action and the migrating complexes are then immobilised and captured on the nitrocellulose matrix of the test strip. Therefore, the more D-dimer antigens in the blood specimen, the more complexes that are accumulated and captured on the test strip and thus signal intensity of fluorescence of detector antibodies reflects the amount of D-dimer captured. Quality control was ensured through a built-in control to ensure accuracy and by minimising false positive results using a specific 3B6/22 monoclonal antibody reagent.

### Statistical analysis

Data were analysed using IBM SPSS statistical software version 28.0 for Windows (Armonk, New York). The normality of continuous data was tested using the Kolmogorov–Smirnov test with Lilliefors’ significance correction. Participants’ demographic and clinical data were summarised in the descriptive statistics as mean (± standard deviation (SD)) for continuous variables and frequency (and percentages) for categorical variables. Univariate analyses were conducted between the participants’ characteristics and the plasma D-dimer categories of the case and comparison groups. Associations between continuous variables were tested using the independent sample *t*-test (normal distribution) or Mann–Whitney *U* and Kruskal Wallis test (skewed data), whereas categorical variables were compared using Pearson’s χ2 or Fisher’s exact test. We performed a receiver operating characteristic (ROC) analysis to select the best discriminating cut-off value of plasma D-dimer that differentiated CC from non-cancer based on optimal sensitivity and specificity. We then tested the associations between plasma D-dimer levels and CC histological prognostic factors such as the type, stage and grade of the disease. Post-hoc analyses were performed to test the difference in the median levels of D-dimer between the different categories of participants in the CC group based on the FIGO stage and histological grade of the disease with significance values adjusted by the Bonferroni correction for multiple tests. Statistical significance was set at *p* < 0.05.

### Statement of ethics

This study protocol was reviewed and approved by the Health Research Ethics Committee of the Lagos University Teaching Hospital with approval number ADM/DCST/HREC/APP/2443 before participants’ enrolment in the study. Ethical principles according to the World Medical Association Declaration of Helsinki were applied throughout the study. The participants were counselled, read and signed an informed consent form before their enrolment in the study. Strict adherence to the privacy and confidentiality of participants’ information was ensured during and after the conduct of the study.

## Results

The mean age of the participants in the CC group (52.8 ± 11.4 years) was not statistically different from that of their cancer-free comparison group (49.3 ± 13.6 years), *p* = 0.120. There were statistically significant differences in parity (*p* = 0.001), tribe (*p* = 0.016), educational level (*p* = 0.001), occupation (*p* = 0.001) and marital status (*p* = 0.047) between the two groups of participants. There were no differences in the age at coitarche (*p* = 0.129), the number of lifetime sex partners (*p* = 0.283), use of oral contraceptive pill (OCP) (*p* = 0.840) and human immunodeficiency virus (HIV) serostatus (*p* = 0.380) between the two groups of participants (Table 1).

**Figure 1.**
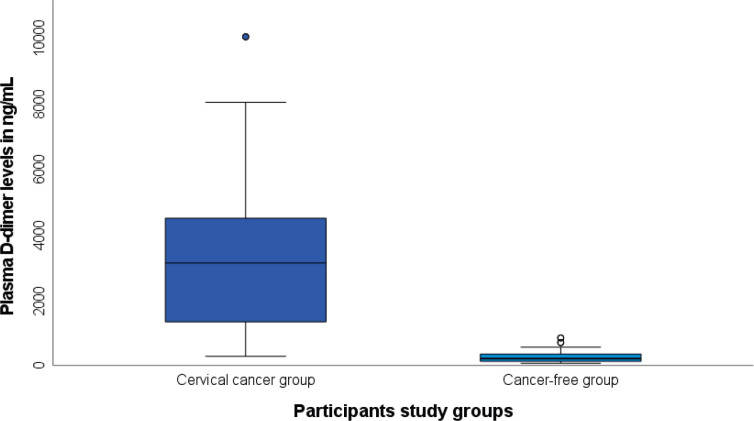
Median plasma levels in women with CC (3,120 (1,189–4,515) ng/mL) versus cancer-free women (210 (125–350) ng/mL), *p* = 0.001.

**Figure 2.**
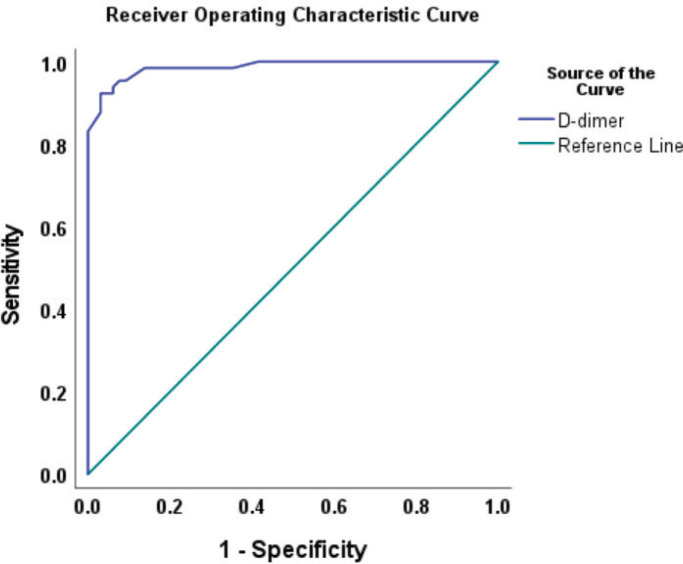
ROC curve showing the best discriminating cut-off value of plasma D-dimer (543 ng/mL) at AUROC curve of 0.986 (95% CI: 0.972, 0.997), *p* = 0.001.

As shown in Figure 1, there was a statistically significant difference in the plasma D-dimer levels between women who had CC, 3,120 (1,189–4,515) ng/mL, and that of their cancer-free counterparts, 210 (125–350) ng/mL, *p* = 0.001. Based on the ROC analysis, the best discriminating cut-off value of plasma D-dimer was 543 ng/mL at an area under the ROC curve (AUROC) of 0.986 (95% confidence interval (CI) 0.972, 0.997), *p* = 0.001 corresponding to a CC detection sensitivity of 95.4% and specificity of 92.3% (Figure 2).

Of the 65 women with CC, the majority had SCC (*n* = 58, 89.2%), early stage I and II (*n* = 44, 67.7%) and histological grade 3 disease (*n* = 25, 38.5%) (Table 2). There were statistically significant associations between plasma D-dimer levels and FIGO stage (*p* = 0.001) and histological grade of CC (*p* = 0.001). Following adjustment by the Bonferroni correction for multiple tests, the post-hoc analyses revealed significant differences in the plasma D-dimer levels between stage I and III (*p* = 0.001), stage I and IV (*p* = 0.001), stage II and III (*p* = 0.008) and stage II and IV (*p* = 0.040). In addition, there were statistically significant differences in the plasma D-dimer levels between grades 1 and 2 (*p* = 0.031) and grades 1 and 3 (*p* = 0.001) (Table 3).

## Discussion

The study was conducted to investigate the possible association between plasma D-dimer levels and CC and its histological prognostic markers among women at a teaching hospital in Lagos, Nigeria. The study found statistically significant associations between elevated plasma D-dimer levels and CC as well as the stage and grade of the disease.

The mean age of women with CC in this study (52.8 ± 11.4 years) is in keeping with that of previous studies conducted among similar cohorts of participants in the same clinical setting [14, 15] and that of the finding by Nkyekyer [16] in a 5-year review of gynaecological cancer patients in Korle Bu Teaching Hospital, Accra, Ghana. This also coincides with the peak age range (50–54 years) at which deaths from CC result in most years of life lost and therefore, it is the time during which the implementation of CC screening would likely be most effective in saving more lives [17]. We found a statistically significant difference between multiparity and CC, a finding which further confirmed the historically documented hypothesis that an increasing number of full-term pregnancies is a significant independent risk predictor of CC [18] due to the increased hormone levels and impaired immune response of pregnancies [19] together with the local tissue damage that occurs during vaginal childbirth resulting in cellular oxidative stress and DNA damage leading to Human papillomavirus (HPV) cellular integration and persistence [20].

There is increasing evidence to suggest that thrombotic episodes may occur months or years long before the diagnosis of cancer [21, 22]. Tumour cells are known to activate the clotting-fibrinolytic system, releasing various fibrinolytic markers and haemostatic factors. These in turn stimulate vascular endothelial cell proliferation and promote neo-angiogenesis necessary for tumour growth [23]. This thus suggests the possible role of thrombotic markers such as plasma D-dimer as potential markers of occult malignancies. The finding of significantly higher median plasma D-dimer levels in women with CC compared with cancer-free women in the current study further corroborates this important theory. This is also in similarity to the finding from the study by Vahid *et al* [24] where higher plasma D-dimer levels were found in malignant gynaecological diseases of the cervix, uterus and ovaries compared to benign lesions and that of a prospective study conducted by Luo *et al* [23] among 296 patients with CC in Guangdong, China. The CC detection sensitivity and specificity of 95.4% and 92.3%, respectively, for the D-dimer assay used in our study suggest its utility as a potential low-cost and convenient diagnostic tool for CC among women in resource-limited settings.

As clotting activation occurs most commonly in cancers, there is a high incidence of thrombosis in metastatic, fast-growing, biologically aggressive cancer with associated poor prognosis [25]. Elevated plasma D-dimer levels, independent of VTE episodes, have been correlated with poorer prognosis of solid gynaecological tumours such as ovarian [21, 26], cervical [23, 27], and endometrial cancer [28]. Preoperative D-dimer has been shown to be an effective prognostic predictor in women with CC [8, 23, 24]. Our current study found significant associations between plasma D-dimer levels and the FIGO stage and grade of CC in close similarity to these previous studies by Li *et al* [8], Luo *et al* [23]and Vahid *et al* [24]. This also corroborates the findings from the study by Nakamura *et al* [27] where pre-operative D-dimer measurement was suggested as a potential biomarker of CC prognosis. In the study by Li *et al* [8], the prognosis of women with CC was poorer if their D-dimer levels were greater than 685 ng/mL which is quite lower than the detection values of greater than 2,235 ng/mL and 3,500 ng/mL for advanced tumour stage (stage III and IV) and poorly differentiated and undifferentiated grade of the disease (high grade), respectively. Although the tumour grade of cervical SCC and adenocarcinoma is regularly included in histopathology reports, at present, there is no grading system that has achieved universal acceptance thus limiting the prognostic value of tumour grade on CC [29]. This is because there are considerable inter-observer variations in the grading of SCC which `subjectively’ depends on how easy it is to recognise the characteristics of squamous epithelium, pleomorphism and mitotic activity [30]. Therefore, as tumour grade is no longer taken into consideration by most guideline recommendations for the management of CC [29, 31, 32], the finding of an association between D-dimer levels and disease grade as reported in this study should be carefully interpreted until future studies are conducted using a standard consensus grading system. Furthermore, the plasma D-dimer levels are not affected by the histological type of CC in this study, thus suggesting its use as a convenient tool for predicting disease prognosis irrespective of the tumour type. A few limitations in our study include the cross-sectional design which made it difficult to infer any causal inferences; the inability to collect accurate historical data on VTE which could confound our findings [33]; and the limited number of participants’ data available for the subgroup analyses.

## Conclusion

We found significant associations between elevated plasma D-dimer levels and CC, and the stage and grade of the disease. The study demonstrated the potential clinical usefulness of plasma D-dimer as a diagnostic and prognostic predictor of CC. However, more reliable evidence should be obtained from future longitudinal studies that will evaluate the longitudinal changes in plasma D-dimer and their relationships to the survival indicators in women with cancerous lesions of the cervix.

## Conflicts of interest

The authors declare that they have no competing interests.

## Funding Information

The funding for this work was mostly provided by the first author (LET) as part of the requirements for the award of his postgraduate fellowship certificate in Obstetrics and Gynaecology. The work was also supported in part by the National Cancer Institute and Fogarty International Center of the National Institutes of Health under Award Numbers K43TW011930, D43TW010934 and D43TW010543. The content of this paper is solely the responsibility of the authors and does not necessarily represent the official views of the National Cancer Institute, Fogarty International Center or the National Institutes of Health.

## Data availability statement

All data generated or analysed during this study are included in this article. Further enquiries can be directed to the corresponding author.

## Figures and Tables

**Table 1.** Characteristics of participants in CC and cancer-free groups^a^.

Characteristic	CC	Non-cancer	*p*-value
	*n* = 65 (%)	*n* = 65 (%)	
Age (y) mean (±SD)	52.8 (±11.4)	49.3 (±13.6)	0.120
Age of coitarche (y) mean (±SD)	18.5 (±4.5)	18.5 (±3.8)	0.917
Parity			0.001^b^
Nulliparous	5 (7.7)	21 (32.3)	
Primiparous	3 (4.6)	2 (3.1)	
Multiparous	57 (87.7)	42 (64.6)	
Tribe			0.016
Yoruba	48 (73.8)	32 (49.2)	
Igbo	11 (16.9)	21 (32.3)	
Others	6 (9.2)	12 (18.5)	
Education			0.001
Uneducated	12 (18.5)	1 (1.5)	
Primary education	14 (21.5)	7 (10.8)	
Secondary education	25 (38.5)	17 (26.2)	
Tertiary education	14 (21.5)	40 (61.5)	
Occupation			0.001
Unskilled	16 (24.6)	12 (18.5)	
Semi-skilled	33 (50.8)	13 (20.0)	
Skilled	8 (12.3)	13 (20.0)	
Professional	8 (12.3)	27 (41.5)	
Marital status			0.047^b^
Married	42 (64.6)	50 (76.9)	
Never married	2 (3.1)	6 (9.2)	
Divorced	9 (13.8)	2 (3.1)	
Separated	1 (1.5)	0 (0.0)	
Widowed	11 (16.9)	7 (10.8)	
Lifetime sex partners			0.283
At most one	23 (35.4)	29 (44.6)	
More than one	42 (64.6)	36 (55.4)	
Previous use of OCP			0.840
Yes	16 (24.6)	17 (26.2)	
No	49 (75.4)	48 (73.8)	
HIV status			0.380
Positive	7 (10.7)	3 (4.6)	
Negative	58 (89.2)	62 (95.4)	

OCP, Oral contraceptive pill; HIV, Human immunodeficiency virus

a Values are given as mean ± SD, or number (percentage) unless indicated otherwise

b Fisher’s exact test

**Table 2.** Tumour characteristics and plasma D-dimer levels in participants with CC (*n* = 65).

Tumour characteristics	Frequency (%)	Plasma D-dimer levels (ng/mL)^a^	*p*-value
Histological type			0.966
SCC	58 (89.2)	3,135 (1,037–4,498)	
Adenocarcinoma	7 (10.8)	2,300 (1,330–4,760)	
FIGO stage			0.001^b^
Stage I	15 (23.1)	840 (590–2,380)	
Stage II	29 (44.6)	2,235 (2,235–3,468)	
Stage III	10 (15.4)	4,760 (4,760–7,350)	
Stage IV	11 (16.9)	5,320 (5,320–6,160)	
Histological grade			0.001^b^
Low grade	19 (29.2)	1,400 (770–2,380)	
Intermediate grade	21 (32.3)	3,500 (1,435–4,655)	
High grade	25 (38.5)	3,780 (2,235–6,580)	

FIGO, International Federation of Gynaecology and Obstetrics; SCC, squamous cell carcinoma

a Plasma levels of D-dimer are given as median (interquartile range)

b Kruskal Wallis test

**Table 3.** Pairwise comparisons of plasma D-dimer levels stratified by FIGO stages and histological grades of CC.

Comparison	Test statistic	Std. error	Std. test statistic	*p*-value	Adjusted *p*-value^a^
FIGO stage					
Stage I and II	−12.661	6.048	−2.093	0.036	0.218
Stage I and III	−34.364	7.503	−4.580	0.001	0.001
Stage I and IV	−30.909	7.503	−4.120	0.001	0.001
Stage II and III	−21.703	6.726	−3.227	0.001	0.008
Stage II and IV	−18.248	6.726	−2.713	0.007	0.040
Stage III and IV	3.455	8.059	0.429	0.668	1.000
Histological grade					
Low and intermediate	−15.330	5.984	−2.562	0.010	0.031
Low and high	−21.471	5.753	−3.732	0.001	0.001
Intermediate and high	−6.141	5.595	−1.098	0.272	0.817

Std., Standard

a Significance values have been adjusted by the Bonferroni correction for multiple tests

## References

[1] Okunade KS, Adejimi AA, John-Olabode SO (2022). An overview of HPV screening tests to improve access to cervical cancer screening amongst underserved populations: from development to implementation. Risk Manag Healthc Policy.

[2] Sung H, Ferlay J, Siegel RL (2021). Global cancer statistics 2020: GLOBOCAN estimates of incidence and mortality Worldwide for 36 cancers in 185 countries. CA Cancer J Clin.

[3] Fitzmaurice C, Dicker D, Global Burden of Disease Cancer Collaboration (2013). The global burden of cancer 2013. JAMA Oncol.

[4] Kim BG (2013). Squamous cell carcinoma antigen in cervical cancer and beyond. J Gynecol Oncol.

[5] Güzel C, van Sten-Van’t Hoff J, de Kok IMCM (2021). Molecular markers for cervical cancer screening. Exp Rev Proteomics.

[6] de Buyzere M, Philippé J, Duprez D (1993). Coagulation system activation and increase of D-dimer levels in peripheral arterial occlusive disease. Am J Hematol.

[7] Gadducci A, Barsotti C, Cosio S (2011). Smoking habit, immune suppression, oral contraceptive use, and hormone replacement therapy use and cervical carcinogenesis: a review of the literature. Gynecol Endocrinol.

[8] Li B, Shou Y, Zhu H (2021). Predictive value of hemoglobin, platelets, and D-dimer for the survival of patients with stage IA1 to IIA2 cervical cancer: a retrospective study. J Int Med Res.

[9] Xu L, He F, Wang H (2017). A high plasma D-dimer level predicts poor prognosis in gynecological tumors in East Asia area: a systematic review and meta-analysis. Oncotarget.

[10] Li W, Tang Y, Song Y (2018). Prognostic role of pretreatment plasma D-dimer in patients with solid tumors: a systematic review and meta-analysis. Cell Physiol Biochem.

[11] Wang X, Ji X (2020). Sample size estimation in clinical research. Chest.

[12] Bhatla N, Berek JS, Cuello Fredes M (2019). Revised FIGO staging for carcinoma of the cervix uteri. Int J Gynaecol Obstet.

[13] Wright JR (2020). Albert C. Broders, tumor grading, and the origin of the long road to personalized cancer care. Cancer Med.

[14] Offor JO, Okunade KS, Iwalokun BA (2021). Evaluation of oxidative markers in women with invasive cervical cancer in Lagos, Nigeria. Ecancermedicalscience.

[15] Sekumade A, Okunade K, Olorunfemi G (2019). Association between serum folate level and invasive cervical cancer at a university teaching hospital in South-West Nigeria. J Cancer Res Pract.

[16] Nkyekyer K (2009). Pattern of gynaecological cancers in Ghana. East Afr Med J.

[17] Law MR, Morris JK, Wald NJ (1999). The importance of age in screening for cancer. J Med Screen.

[18] Okunade KS (2019). Human papillomavirus and cervical cancer. J Obstet Gynaecol.

[19] International Collaboration of Epidemiological Studies of Cervical Cancer (2006). Cervical carcinoma and reproductive factors: collaborative reanalysis of individual data on 16,563 women with cervical carcinoma and 33,542 women without cervical carcinoma from 25 epidemiological studies. Int J Cancer.

[20] Williams VM, Filippova M, Soto U (2011). HPV-DNA integration and carcinogenesis: putative roles for inflammation and oxidative stress. Future Virol.

[21] Man YN, Wang YN, Hao J (2015). Pretreatment plasma D-dimer, fibrinogen, and platelet levels significantly impact prognosis in patients with epithelial ovarian cancer independently of venous thromboembolism. Int J Gynecol Cancer.

[22] Chen WH, Tang LQ, Wang FW (2014). Elevated levels of plasma D-dimer predict a worse outcome in patients with nasopharyngeal carcinoma. BMC Cancer.

[23] Luo YL, Chi PD, Zheng X (2015). Preoperative D-dimers as an independent prognostic marker in cervical carcinoma. Tumor Biol.

[24] Vahid Dastjerdi M, Ahmari S, Alipour S (2015). The comparison of plasma D-dimer levels in benign and malignant tumors of cervix, ovary and uterus. Int J Hematol Oncol Stem Cell Res.

[25] de Buyzere M, Philippé J, Duprez D (1993). Coagulation system activation and increase of D-dimer levels in peripheral arterial occlusive disease. Am J Hematol.

[26] Sakurai M, Satoh T, Matsumoto K (2015). High pretreatment plasma D-dimer levels are associated with poor prognosis in patients with ovarian cancer independently of venous thromboembolism and tumor extension. Int J Gynecol Cancer.

[27] Nakamura K, Nakayama K, Ishikawa M (2016). High pre-treatment plasma D-dimer level as a potential prognostic biomarker for cervical carcinoma. Anticancer Res.

[28] Li J, Lin J, Luo Y (2015). Multivariate analysis of prognostic biomarkers in surgically treated endometrial cancer. PLoS One.

[29] McCluggage WG (2018). Towards developing a meaningful grading system for cervical squamous cell carcinoma. J Pathol Clin Res.

[30] Graflund M, Sorbe B, Hussein A (2002). The prognostic value of histopathologic grading parameters and microvessel density in patients with early squamous cell carcinoma of the uterine cervix. Int J Gynecol Cancer.

[31] Eifel PJ, Fisher CM, Frederick P (2017). NCCN Guidelines ® Version 1.

[32] Cibula D, Pötter R, Planchamp F (2018). The European society of gynaecological oncology/European society for radiotherapy and oncology/European society of pathology guidelines for the management of patients with cervical cancer. Virchows Archiv.

[33] Arpaia G, Carpenedo M, Verga M (2009). D-dimer before chemotherapy might predict venous thromboembolism. Blood Coagul Fibrinolysis.

